# Terrible APS — a newly defined variant of severe APS

**DOI:** 10.3389/fimmu.2025.1721515

**Published:** 2026-01-07

**Authors:** Stanley Niznik, Tania Zaher, Soad Haj Yehia, Ronen Shavit, Shiri Weinstein, Yulia Lifshitz, Nancy Agmon-Levin

**Affiliations:** 1 Clinical Immunology, Angioedema and Allergy Unit, Sheba Medical Center, Ramat Gan, Israel; 2 School of Medicine, St. Georges University of London, London, United Kingdom; 3 Sackler School of Medicine, Tel Aviv University, Tel Aviv, Israel

**Keywords:** antiphospholipid syndrome (APS), microvascular disease, heart valve disease, anti-phospholipid antibodies, traps

## Abstract

**Introduction:**

Antiphospholipid syndrome (APS) presents with various clinical features and some patients exhibit progressive, refractory disease that does not meet the catastrophic APS (cAPS) criteria. This study describes a new subcategory of APS patients, “Terrible APS” (TrAPS), characterized by recurrent thrombosis despite optimal anticoagulation, often requiring immunomodulation or surgery.

**Methods:**

We analyzed 306 primary APS patients, excluding those with obstetric, non-criteria APS, or cAPS. TrAPS was defined as >2 breakthrough thrombotic events despite anticoagulation (without provocation or cardiovascular risk) or the need for >1 immunomodulatory or surgical intervention.

**Results:**

Among 209 patients with thrombotic primary APS, 27 (12.7%) met the TrAPS criteria. These patients had higher rates of venous thrombosis, microvascular involvement, heart valve disease, thrombocytopenia, and triple-positive antiphospholipid antibodies. TrAPS was associated with *increased mortality* (18.5% *vs*. 5.1%) and anticoagulant resistance (81.4% with breakthrough events). Based on multivariate analysis, we identified four key predictors that formed the basis of the TrAPScore: severe thrombocytopenia (<50,000, 4 points), heart valve involvement (4 points), microvascular manifestations (3 points), and triple-positive serology (2 points). A TrAPScore >6 had a positive predictive value (PPV) of 78%–82.5%, while a score <4 had a negative predictive value (NPV) of 96.9% for TrAPS diagnosis.

**Conclusion:**

We herein individualized a particularly refractory APS subcategory, TrAPS. The TrAPScore incorporates severe thrombocytopenia, heart valve disease, microvascular manifestations, and triple-positive serology. A TrAPS score >6 predicted a high likelihood of severe, refractory disease while effectively excluding TrAPS.

## Introduction

Antiphospholipid syndrome (APS) is initially characterized by its association with thrombosis (e.g., stroke), obstetric complications, and the presence of lupus anticoagulant (LAC) (1, 2). Over time, this immune-mediated condition was further linked to a myriad of clinical manifestations, including macro- and microvascular thrombotic events, obstetric morbidity, heart valve disease, thrombocytopenia, and hemolytic anemia. Additionally, immunoassays used to detect antiphospholipid antibodies have evolved to include anti-cardiolipin (ACL) and anti-beta-2 glycoprotein (aB2GPI) autoantibodies alongside LAC (2–4). The classification criteria for APS have been revised several times and were established at the Sapporo summit 2006 (5) and recently redesigned in the new classification published in 2023 by the American College of Rheumatology and the European League Against Rheumatism (ACR-EULAR). The latter expanded the clinical criteria and refined the parameters for positive antibody assays (6). Furthermore, therapeutic guidelines published by EULAR emphasize the importance of homeostatic control in association with severity of disease as well as the role of immunomodulation with antimalarial drugs (7).

Despite improved understanding, definitions, and designed treatment protocols, clinicians still struggle, as the recurrence rate of APS manifestations ranges from 30% to 50% during long-term follow-up (8, 9). Moreover, APS disease progression is often unpredictable and may be severe or even catastrophic. Several decades ago, a very small subset of APS that manifests mainly with acute multiple microvascular thrombosis across different vascular beds within a short period, was defined as catastrophic APS (cAPS). This catastrophic variant is associated with high morbidity and mortality rates and requires intensive multifaceted interventions that have been found to be beneficial (10, 11). However, APS may present as a severe disease that is more gradual and does not fit the definition of cAPS. Severe APS appears to be more prevalent than cAPS and is less well-defined. In this line of thought, two APS severity scores were developed and validated: the adjusted Global Antiphospholipid Syndrome Score (aGAPSS) and the Damage Index for Thrombotic Antiphospholipid Syndrome (DIAP) (12–16). Additionally, some studies have investigated specific APS manifestations (i.e., heart valve disease or thrombocytopenia) as predictors of a worse disease course (17–19). Nonetheless, there is a lack of data on how to define severe and very severe APS, as well as the urgent need to improve disease outcomes, which potentially require a more aggressive approach and/or the addition of immunomodulation.

Hence, in this study we aim to identify a new variant of APS disease, which on the one side does not fit the cAPS definition yet on the other is extremely severe and refractory to current recommended therapy. We further aimed to understand what distinguishes this very severe variant and explore the possibility of developing a diagnostic tool to enable early detection of this variant, which may enable the study of tailored treatments for patients who present with this variant.

## Objective

In this study, our objective was to define a specific subset of thrombotic antiphospholipid syndrome (APS) that is resistant, gradually progressive, and does not fit the criteria for catastrophic antiphospholipid syndrome (non-cAPS) (20). In addition, we aimed to identify key parameters associated with this very severe subgroup of APS and to develop a statistical tool that can assist clinicians in identifying patients at risk.

## Methods

### Patients

We enlisted all patients diagnosed with primary APS who were actively followed at our center between 2011 and 2024, and those who met the inclusion criteria for this study were included in the final analysis. Data were collected retrospectively to analyze demographic, clinical, and serological parameters, as well as details regarding disease progression. This study was performed in accordance with the Declaration of Helsinki and approved by the Sheba Medical Center Review Board.

### Inclusion criteria

• Antiphospholipid syndrome (APS) was diagnosed according to the revised Sapporo Criteria (5).

• Primary APS patients—At Selection, all patients were diagnosed with primary antiphospholipid syndrome. A secondary autoimmune disease was documented if it developed during follow-up.

• Active regular follow-up (e.g., >1 follow-up visit within 12 months).

### Exclusion criteria

• Catastrophic APS diagnosis according to the international consensus (10, 20).

• Non thrombotic disease (e.g., pure obstetric APS).

• Secondary APS at diagnosis.

• Non-criteria APS (e.g., not fulfilling the revised Sapporo APS criteria).

### Clinical manifestations

We monitored APS-related events (e.g., thrombocytopenia and valve disease), specifically APS recurrences, including both thrombotic and non-thrombotic events. Notably, *breakthrough thrombotic events* were distinct from recurrent thrombotic events despite the use of full anticoagulant therapy (excluding DOAC therapy), absence of cardiovascular risk factors, and, in the case of venous thrombosis, absence of a provocation. *Microvascular involvement* was defined as the presence of one or more of the following: renal failure or proteinuria (with no other explanation), diffuse alveolar hemorrhage, or myocardial infarction without occlusive disease; a composite score of which any of the previous was positive, named any microvascular presentation. Microvascular involvement and other non-criteria manifestations were assessed at any time during follow-up.

We assessed the therapies required, including antiaggregation, anticoagulation, and immunomodulation, alongside any surgical interventions related to APS, mainly vascular procedures and heart valve repair or replacement. We also used the validated aGAPSS, which allots three points for dyslipidemia, one for arterial hypertension, five for anti-cardiolipin antibodies IgG/IgM, four for anti-β2 glycoprotein IgG/IgM, and four for lupus anticoagulant (12). We also calculated the proposed adjusted Global Antiphospholipid Syndrome Score for Cardiovascular Disease (aGAPSS-CVD), which incorporates factors such as smoking, diabetes, and obesity. This score emphasizes the risk of cardiovascular disease in patients with APS (21).

### Serology

The presence of anti-cardiolipin (aCL) and anti-β_2_‐glycoprotein I (aβ_2_GPI) IgG and IgM isotypes was measured using enzyme‐linked immunosorbent assay (ELISA) or a multiplex system. The kits that were used were all commercial (ELISA—aB2GPI by AESKU Diagnostics (Wendelsheim, Germany) and aCL by Varelisa (Pharmacia Diagnostics, Stockholm, Sweden); Bioplex both aB2GPI and aCL (Bio-Rad, Hercules, CA, USA). B2GPI and ACL were considered positive if antibody levels were above 20 MPL units (IgG phospholipid units or IgM phospholipid units), or if >99th percentile or according to the manufacturer’s instructions were present in a minimum of two tests performed at least 12 weeks apart.

Lupus anticoagulant (LA) activity was detected using coagulation assays routinely performed at each center, consistent with the International Society of Thrombosis and Hemostasis guidelines (22). The LA assays were modified in 2016; until 2016, LA activity was measured by LA-responsive activated partial thromboplastin time (aPTT) aPL (by Stago, confirmed using the Actem FS Kit by Siemens, Erlangen, Germany), and from 2016, LA activity was measured by a combination of silicon clotting time and the use of the Russell Viper Venom Kit (IL, Bedford, MA, USA). In the case of anticoagulation treatment or spontaneous INR >1.5, patients’ plasma was mixed with normal plasma to reduce false positivity. Positivity was defined as single, double, or triple positivity according to the number of positive tests.

### APS severity definition

In this study, three independent researchers reviewed all medical files and scored each patient’s disease as Mild, Intermediate, or Severe. If inconsistencies were documented between researchers, the patient file was re-reviewed in a joint meeting to reach a consensus. Notably, patients with Catastrophic APS (as per the definition of the CAPS registry group (10)) at any time during the disease were excluded. In addition, we compared patients with TrAPS to those with mild or intermediate disease.

Risk stratification:

A. Mild APS disease

- No recurrence of APS manifestations.
- Thrombotic recurrence while treated with anti-aggregating therapy only, but no recurrence once switched to anticoagulation therapy.
- Recurrence due to cessation of therapy and/or poor adherence to therapy.
- No need for immunomodulation therapy other than hydroxychloroquine.

B. Intermediate APS disease (did not meet mild or severe criteria)

- Thrombotic events post APS diagnosis, including any of the following:
  ◼ Transient ischemic attack with no lesions on magnetic resonance imaging.
  ◼ Arterial thrombotic events with frank cardiovascular risk factors (i.e., hypertension, dyslipidemia, smoking, or diabetes mellitus).
  ◼ Venous recurrence while patients on low dose anti-coagulation therapy (e.g., low molecular weight heparin 0.5 mg/kg once daily).
  ◼ Thrombotic recurrence (either arterial or venous) while treated with direct oral anticoagulants.
  ◼ Use of immunomodulation with low-dose glucocorticoids and/or cDMARDs (e.g., azathioprine) only.
  ◼ Surgical interventions due to APS manifestations while non-adherence to anticoagulation therapy.

Severe APS disease (at least one of the following):

- ≥1 *Breakthrough Events*
  ○ or
- Surgical intervention (e.g., need for limb amputation) due to APS despite treatments according to the current guidelines.
  ○ or
- APS manifestations that required treatment with one advanced immunomodulation (i.e., rituximab, intravenous immunoglobulin, or plasma exchange therapy).

Terrible APS (TrAPS) variant was defined if:

- >2 *Breakthrough Events*
  or
- >1 surgical intervention (e.g., pulmonary endarterectomy, heart valve surgery, and/or limb amputation, etc.).
  ○ or
- APS manifestation that required treatment with >1 with advanced immunomodulation (i.e., combination of rituximab, intravenous immunoglobulin, or plasma exchange therapy, etc.).

### Statistical analysis

The data were analyzed using jamovi version 2.6 [Computer Software], developed by The Jamovi Project (2025). This can be accessed at https://www.jamovi.org. To analyze between-group differences in discrete variables, either Pearson’s chi-square test or Fisher’s exact test (two-tailed) was employed. Variables that were found to be significant (p <0.10) in the univariate analysis were subsequently used in a stepwise logistic regression to identify those most significantly associated with each outcome measure. The selection of variables for the stepwise analysis was limited based on the total number of patients, allowing for one variable for every five patients in the study. Patients with missing data were excluded from the analysis. Odds ratios (ORs) and 95% confidence intervals (CIs) were calculated, with a significance level set at p ≤0.05.

## Results

**Figure 1:**
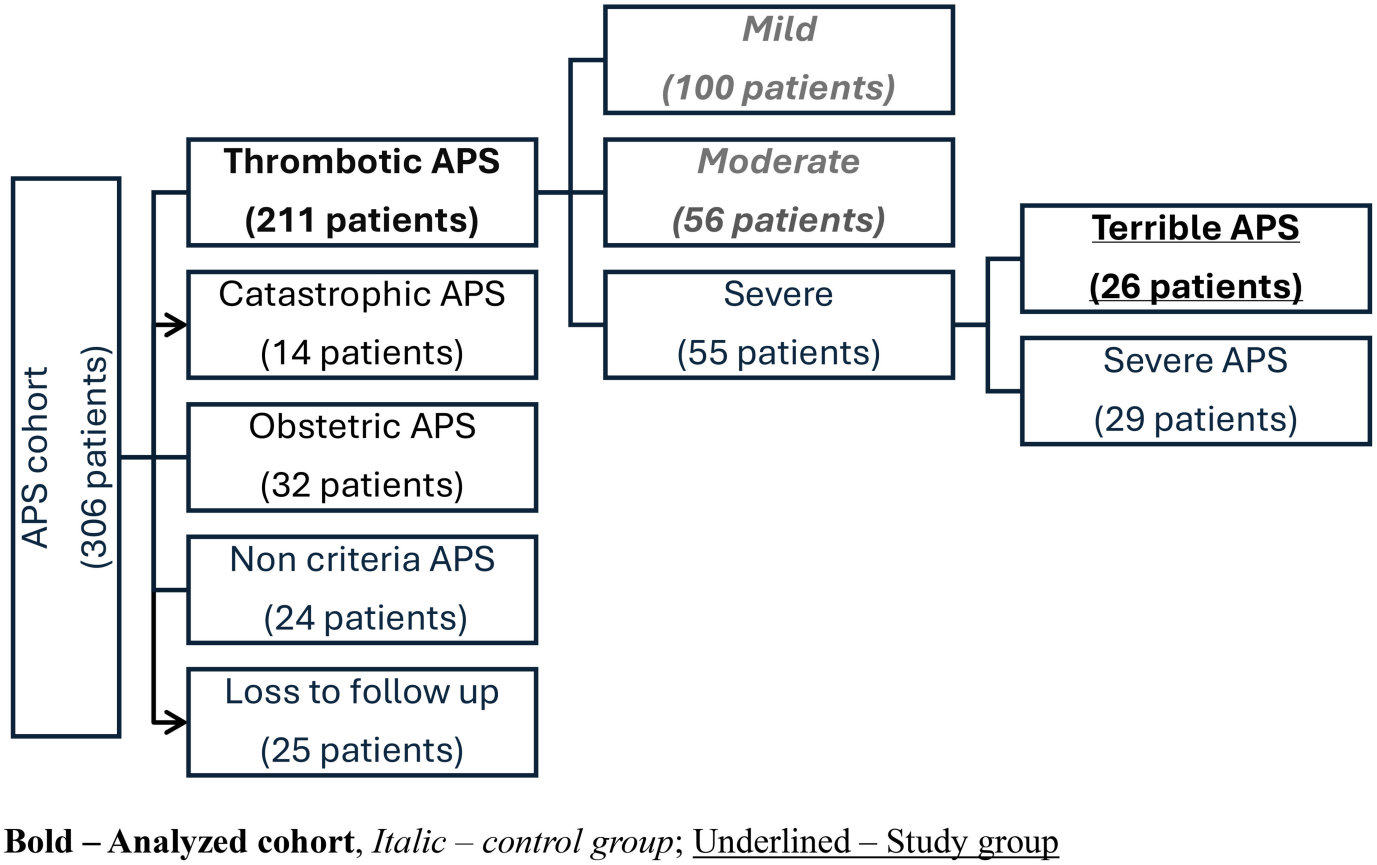
Our cohort of APS patients. Bold—Analyzed cohort, *Italic—control group*; Underlined—Study group.

In this study, 306 patients with primary APS were assessed, and upon review and exclusion based on our study criteria, 209 patients with thrombotic APS were designated as suitable for severity stratification. Of these, study group included 27 of 209 (12.7%) patients with the new variant TrAPS, compared to a control group of 156 patients, a composite of mild and moderate APS disease (**Figure 1**).

### Demography, cardiovascular risk factors, and APS presenting manifestations of TrAPS *vs*. APS controls

Our study and control groups were similar in terms of age, sex, and cardiovascular risk factors (**Table 1**). The rate of additional autoimmune disease diagnosis during follow-up was low (5%–10%) and did not differ between the groups. Notably, the follow-up time among patients with TrAPS was longer, with an average of 4.9 years. Additionally, venous thrombosis at presentation was associated with the diagnosis of TrAPS during follow-up. Obstetric morbidity at presentation was inversely associated with TrAPS, although this difference did not reach statistical significance. Interestingly, in the TrAPS group, five patients upon presentation had signs of both venous thrombosis and arterial thrombosis (ex. signs of pulmonary embolism on CTA in the stroke protocol or signs of ischemic events on brain imaging during patient evaluation for pulmonary embolism), this patient did not fulfil the criteria for CAPS.

**Table 1:** Demographics and presenting symptoms of TrAPS *vs*. APS-control groups.

Parameter	Terrible APS (n = 26)	APS control group (n = 156)	P value
Gender (Female)	20 (76.9%)	110 (70.8%)	0.708
Age at presentation	34 (CI95% 32–40.8)	38 (CI95% 30.1–45.9)	0.199
Follow up period (years)	16 (CI95% 10.3–18.45)	11.1 (CI95% 6.6–15.6)	0.012
Secondary APS diagnosis during follow-up	1 (3.8%)	16 (10.2%)	0.711
Hypertension	6 (23.1%)	41 (26.2%)	0.658
Smoking	5 (19.2%)	30 (19.2%)	0.588
Dyslipidemia	6 (23.1%)	35 (22.4%)	0.981
Diabetes Mellitus	2 (7.7%)	12 (7.7%)	0.959
Obesity	8 (30.7%)	29 (18.8%)	0.186
Venous thrombosis at presentation^*^	15 (57.7%)	45 (28.8%)	0.006
Arterial Thrombosis at presentation	16 (61.5%)	78 (50%)	0.21
Obstetric morbidity at presentation (female only)	4 (14.8%)	50 (32%)	0.07

^*^Five patients upon presentation had signs of both venous thrombosis and arterial thrombosis.

### Laboratory findings and serology

The diagnosis of TrAPS was linked to the presence of triple aPL positivity, which was mostly evident for anti-β2-Glycoprotein I and anti-cardiolipin of the IgG subtypes. Interestingly, anti-cardiolipin antibodies of the IgM isotype (ACL IgM) were inversely correlated with TrAPS. In addition, APS-associated thrombocytopenia of any severity was linked to TrAPS (data not shown), while extreme thrombocytopenia (<50,000) was highly prevalent in our study group, as well as clinical evidence of both hemolytic anemia and thrombocytopenia, defined as Evans syndrome (**Table 2**).

**Table 2:** Laboratory parameters of TrAPS *vs*. APS-control groups.

Parameter	Terrible APS (n = 26)	APS control group (n = 156)	P value
Anti B2 Glycoprotein IGG positivity	24 (92.3%)	96 (69.5%)	0.005
Average B2 Glycoprotein IgG Titer (u/ml)	131 (± 56.3)	68.6 (± 67.3)	<0.001
Anti B2 Glycoprotein IgM positivity	12 (46.1%)	75 (48%)	0.727
Average B2 Glycoprotein IgM Titer (u/ml)	35.5 (± 48.7)	38.7 (± 46.8)	0.725
Anti Cardiolipin IgG positivity	24 (92.3%)	80 (51.2%)	<0.001
Average Anti Cardiolipin IgG Titer (u/ml)	138 (± 51.2)	70.8 (± 68.3)	<0.001
Anti Cardiolipin IgM positivity	6 (23.1%)	70 (44.9%)	0.027
Average Anti Cardiolipin IgM Titer (u/ml)	25.9 (± 43.7)	31.2 (± 39.9)	0.207
Lupus anticoagulant positivity	25 (96.1%)	114 (73.1%)	0.028
Triple Positive aPLs	24 (92.3%)	80 (51.2%)	<0.001
Anti-nuclear Ab. (ANA)	16 (61.5%)	88 (56.4%)	0.886
Direct Coombs test positivity	9 (34.6%)	30 (19.2%)	0.07
Evans syndrome*	6 (23.1%)	10 (6.4%)	0.007
Severe thrombocytopenia (<50,000/µL)	7 (26.9%)	4 (2.5%)	<0.001
C3 mg/dL (Average nadir)	76.5 (CI95% 64.7–89.3)	100.6 (CI95% 92.6–120.3)	0.35
C4 mg/dl (Average nadir)	19.2 (CI95% 13.8–24.5)	23 (CI95% 17.7–28.4)	0.83

*Autoimmune hemolytic anemia and thrombocytopenia.

### Microvascular, heart valve involvement, and non-criteria manifestation

Nephropathy, diffuse alveolar hemorrhage, and myocardial disease were significantly more common in the TrAPS group, whereas livedo reticularis and other non-criteria manifestations (skin ulcers, migraine, and epilepsy) did not differ from those in the control group (**Table 3**).

**Table 3:** Microvascular, heart valve, and non-criteria manifestations among TrAPS *vs*. APS control groups.

Parameter	Terrible APS (n = 26)	APS control group (n = 156)	P value
Any microvascular =	18 (69.2%)	11 (7.1%)	<0.001
* Nephropathy*	*11 (42.3%)*	*22 (14.1%)*	*<0.001*
* Diffuse alveolar hemorrhage*	*8 (30.8%)*	*0 (0%)*	*<0.001*
* Myocardial disease with normal coronaries*	*4 (15.3%)*	*1.2%*	*<0.001*
* Adrenal hemorrhage*	*1 (3.8%)*	*0 (0%)*	*0.016*
Heart Valve Disease	16 (61.5%)	19 (12.2%)	<0.001
* Thickening*	*13 (50%)*	*15 (9.6%)*	*<0.001*
* Vegetation*	*8 (30.8%)*	*7 (4.5%)*	*<0.001*
Livedo reticularis	3 (11.5%)	20 (12.8%)	0.806
Skin ulcer	3 (11.5%)	9 (5.7%)	0.377
Migraine	3 (11.5%)	28 (17.9%)	0.377
Epilepsy	4 (15.3%)	14 (9%)	0.091

### APS disease outcomes and treatments

Mortality was almost triple in the TrAPS group, which was statistically significant (**Table 4**). Focusing on the causes of death in the control group, three individuals died from malignancies, three from complications related to cerebrovascular accidents (CVA), one from COVID-19, and one from sepsis. In contrast, all causes of death in the TRAPS group were linked to APS morbidity rather than other disease processes, reflecting the aggressive and refractory course of this condition.

**Table 4:** Mortality and APS outcomes among TrAPS group *vs*. APS controls.

Parameter	Terrible APS (n = 26)	APS control group (n = 156)	P value
Death of any cause	5 (19.2%)	8 (5.1%)	0.012
Death d/t APS event	5 (19.2%)	3 (1.9%)	<0.001
Arterial thrombosis recurrence	14 (53.8%)	44 (28.2%)	0.014
Venous thrombosis recurrence	15 (57.7%)	23 (14.7%)	<0.01
≥2 breakthrough thrombotic recurrence ^*^	16 (61.5%)	0 (0%)	<0.01
>1 APS associated surgical intervention	5 (19.2%)	0 (0%)	<0.01
Requirement for treatments with >1 immunomodulation ^**^	9 (34.6%)	0 (0%)	<0.01
Any APS related surgical intervention^#^	19 (73.1%)	8 (5.1%)	<0.01
Heart valve replacement	7 (26.9%)	3 (1.9%)	<0.01
Vascular intervention (Thrombectomy Endarterectomy, Digit or limb amputation)	13 (50%)	5 (3.2%)	<0.01
Obstetric morbidity recurrence	3 (11.6%)	39 (35.4%)	0.07
aGAPSS^$^	12.4 (CI95% 11.4-13.4)	11.1 (CI95% 9.4-12.8)	0.001
aGAPSScvd^$$^	14.3 (CI95% 13.1-16.2)	11.8 (CI95% 10-12.4)	0.001

^*^A thrombotic event despite full anticoagulant therapy with and if arterial thrombotic recurrence no preexisting cardiovascular risk factors.

^**^APS manifestation that required treatment with >1 with advanced immunomodulation (i.e., combination of rituximab, intravenous immunoglobulin, or plasma exchange therapy).

^#^Composite group of patients required either heart valve replacement or vascular intervention.

^$$^average adjusted Global APS score.

^$$^average adjusted Global APS score cardiovascular.

Given the inclusion criteria and research methodology, it is not surprising that the rate of thrombotic recurrence was higher in the TrAPS group. Even milder forms of APS exhibit a significant recurrence rate despite anticoagulant treatment, with more than 80% of patients within the TrAPS group experiencing this type of recurrence (**Table 4**). Particularly, breakthrough events—defined as thrombosis despite full anticoagulant therapy (DOAC therapy excluded), with no other cardiovascular risk factors—were present only in the TrAPS group (per design), as 18/27 (66%) patients had two or more such events (**Table 4**). Nonetheless, both aGAPSS and aGAPSS-CVD scores were significantly higher in the TRAPS group, confirming their validity as tools for assessing the severity of antiphospholipid syndrome (APS).

In this study, treatments, either surgical or medical, were according to the treated physician dissertation. The TrAPS group was particularly resistant to anticoagulant therapy, and 81.4% (22 of 27 patients) of patients with TrAPS required significant adjunctive therapy. Of which 2/3 of patients with TrAPS required multiple types of immune modulation (i.e., two or more of IVIG, rituximab, or plasmapheresis) to achieve disease control. Moreover, 19/27 (70.3%) patients with TrAPS required surgical intervention, while 18.5% (5/27) required multiple surgical interventions (**Table 4**).

### Multivariate analysis and TrAPScore design

In our univariate analysis, we found that TrAPS was associated with venous thrombosis at presentation, longer follow-up, microvascular manifestations, triple aPLA positivity (particular IgG subtypes, both in positivity and titter, as well as the presence of lupus anticoagulant), thrombocytopenia, and Evans syndrome. In comparison, obstetric morbidity at presentation and IgM aCL antibodies were associated with less severe disease.

**Figure 2:**
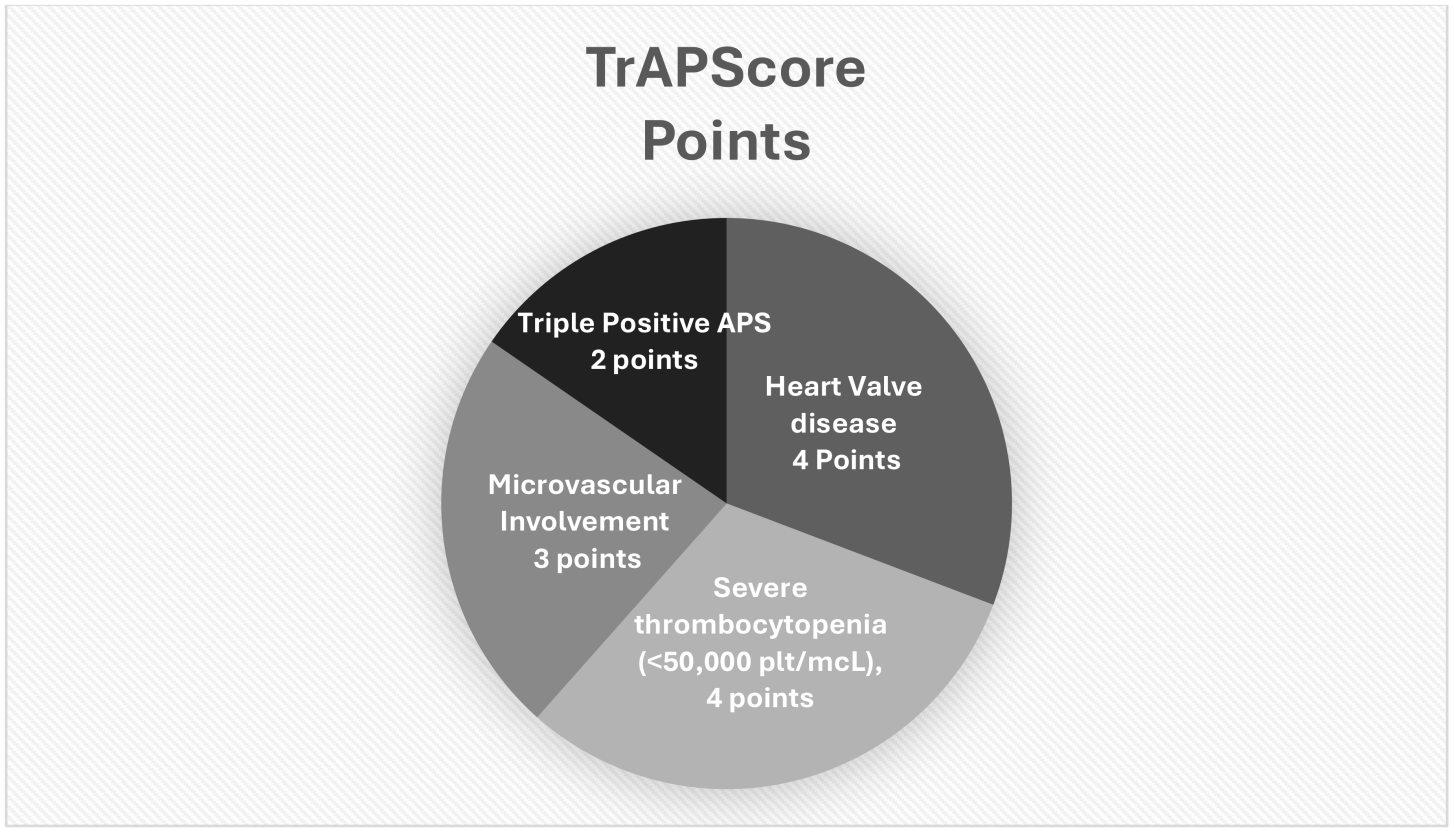
Visual figure of the TrAPScore.

To better understand which parameters correlated most with the TrAPS group, we performed a multivariate analysis, in which four parameters proved to be the most significant (area under the curve, 0.902). Based on this ratio, we developed the TrAPScore (**Figure 2**).

- Severe thrombocytopenia OR 9.7 (CI95% 1.55–60.8) TrAPScore 4 points.
- Heart valve disease OR 8.8 (CI95% 2.9–26) TrAPScore 4 points.
- Microvascular involvement OR 7.1 (CI95% 2–21) TrAPScore 3 points.
- Triple positive aPLs OR 5.5 (CI95%1.4–22.9) TrAPScore 2 points.

To provide cutoffs for the TrAPScore that may enable the prediction of TrAPS, the TrAPScore was evaluated across different comparisons. When distinguishing the TrAPS group (n = 27) from the APS control group (n = 156) using a cutoff score >6, the test demonstrated a sensitivity of 51.4% and a high specificity of 97.4%. The positive predictive value (PPV) was 77.8% and the negative predictive value (NPV) was 92.1%, with an overall accuracy of 90.7%. Based on this analysis, we concluded that TrAPScore >6 is a plausible cutoff for defining TrAPS.

**Figure 3:**
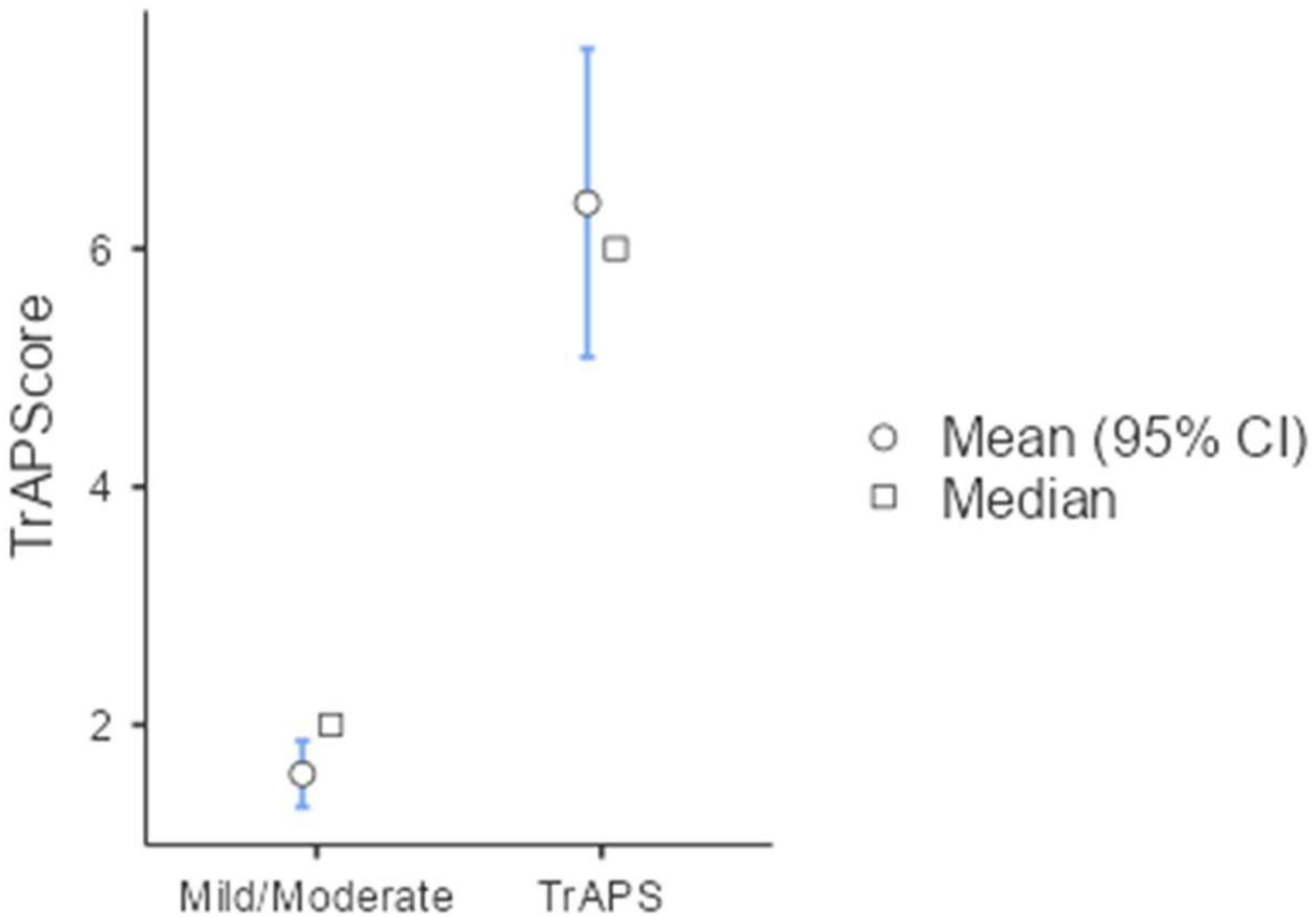
TrAPS score mild/moderate and TrAPS.

To establish an appropriate cutoff for a negative TrAPScore, we determined that a TrAPScore <4 effectively differentiated the TrAPS group from the APS control group. At this threshold, the sensitivity was 85.2%, and the specificity was 79.5%. The positive predictive value (PPV) was 41.8%, whereas the negative predictive value (NPV) was notably high at 96.9%. The overall accuracy of this cutoff was 80.3%. A TrAPScore of less than 4 thus demonstrates a high NPV of 96.9%, suggesting that it could serve as a reliable exclusion criterion for diagnosing this APS variant (**Figure 3**).

### TrAPS and severe APS

Based on the study design, we initially compared TrAPS with mild or moderate disease. To better understand whether TrAPS is distinguishable from other forms of APS, we compared the clinical features of severe APS (**Table 5**). The groups exhibited less pronounced differences, with notable variations primarily observed in the components of the TrAPS score, namely, heart valve disease, microvascular involvement, and triple positivity. Although severe thrombocytopenia was more common in the TrAPS group, this difference was not statistically significant. Additionally, mortality rates were nearly twice as high in the TrAPS cohort; however, this difference was not statistically significant.

**Table 5:** Comparison between TrAPS and severe APS phenotype.

Parameter	Terrible APS (n = 26)	Severe APS (n = 29)	P value
Age at presentation	34 (CI95% 28.3–39.7)	33.1 (CI95% 28.3–39.7)	0.797
Sex (% of females)	19 (73.1%)	19 (65.5%)	0.83
Hypertension	6 (23.1%)	9 (31%)	0.517
Smoker	4 (15.3%)	9 (27.5%)	0.283
Diabetes mellitus	2 (7.6%)	2 (6.9%)	0.912
Dyslipidemia	6 (23.1)	6 (20.6%)	0.83
Venous thrombosis at presentation	15 (57.7%)	18 (62.2%)	0.946
Arterial thrombosis at presentation	16 (61.5%)	11 (37.9%)	0.04
Triple Positive aPLs	24 (92.3%)	19 (65.5%)	0.047
Thrombocytopenia	18 (69.2%)	13 (44.8%)	0.071
Severe thrombocytopenia (<50,000/µL)	7 (26.9%)	3 (10.3%)	0.11
Any microvascular involvement	18 (69.2%)	6 (20.7%)	<0.001
Heart Valve diseease	16 (61.5%)	9 (31%)	0.023
aGAPSS	13.4 ( ± 2.39)	11.7 ( ± 3.71)	0.047
Death of any cause	5 (19.2%)	3 (10.3%)	0.409

**Figure 4:**
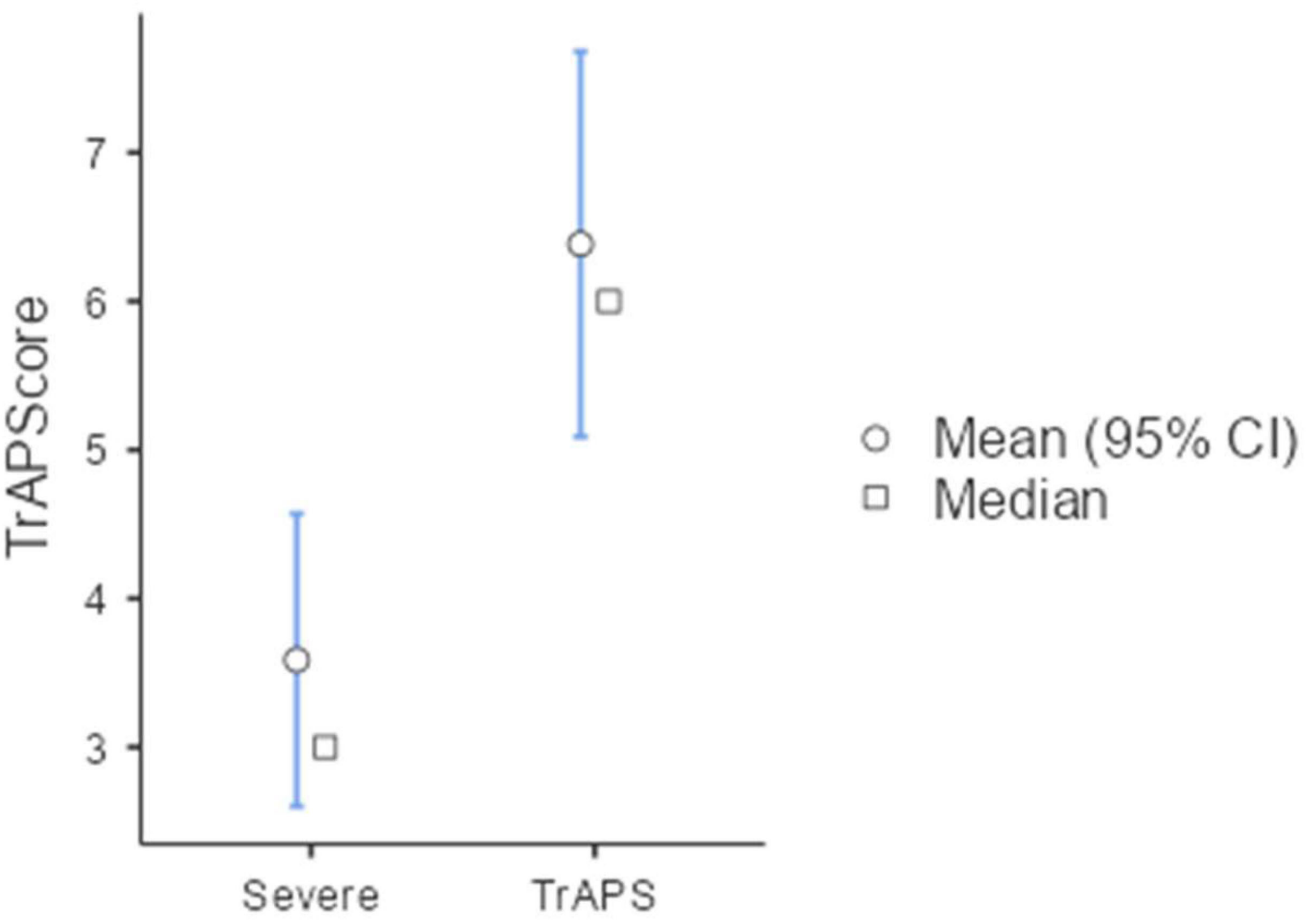
TrAPScore in severe APS *vs* TrAPS.

In the comparison between the TrAPS group (n = 27) and the severe APS group (non-TrAPS; n = 28), the same cutoff yielded a sensitivity of 51.8%, specificity of 88%, PPV of 82.5%, NPV of 63.5%, and overall accuracy of 69% (**Figure 4**).

## Discussion

In this study, we aimed to provide a new perspective on APS severity by defining a novel variant with a particular focus on long-term resistance to recommended therapies. This aim arises from the patient’s bedside, as resistant APS is not an infrequent challenge. Notably, TrAPS differs from catastrophic-APS in terms of both the time frame and pattern of manifestations. Nonetheless, TrAPS like cAPS, may require a more aggressive approach to control.

The notion of better defining APS severity and/or APS risk stratification stands in agreement with several previous attempts that resulted in compelling and well-validated scores (13, 15). One might wonder why another risk stratification is needed, underscoring the focus of TrAPS inception to address the unmet need to define a new clinical tool for therapy-resistant disease. In this line of thought, several recent publications have focused on the use of immune modulating agents, particularly in resistant APS (23–25). Others have tried to focus on the clinical aspects that would be resistant to anticoagulant therapy (26). A recent review of new treatment targets deemed immunomodulatory therapies to be reserved for those with refractory disease and microvascular and non-thrombotic manifestations of APS (27). The goal of this study was to address and this unmet need.

Hence, we selected a homogeneous group of primary thrombotic APS patients, excluding secondary APS, non-thrombotic patients (i.e., OAPS), and non-criteria diseases. All patients were under active follow-up by a dedicated team in our APS center, which enabled a thorough assessment of disease progression, compliance with treatment, and evaluation of treatment outcomes. We applied specific objective criteria, including the definition of breakthrough thrombotic events despite full anticoagulant therapy for arterial thrombosis, the absence of other cardiovascular risk factors, and the lack of appropriate provocation for venous thrombosis, all of which have been detailed in the new classification criteria for APS (6). We also considered other markers of resistance to therapy, such as the need for immune modulation (such as anti-CD20 therapy, IVIG, or plasma exchange) and/or surgical interventions (e.g., heart valve replacement or vascular surgery) related to APS.

In this cohort, 27/209 (12.7%) of the thrombotic APS patients were included in the TrAPS group. This unique group had a specific profile at presentation, which was associated with venous thrombosis, higher aGAPSS score, and the presence of aPLs of the IgG subtype. In contrast, obstetric morbidity and aPLs of the IgM subtype may be considered “protective,” suggesting an interesting role for different aPLs in APS pathogenesis, which remains to be elucidated. As presumed outcomes of the TrAPS group were worse, mortality was higher (18.5% *vs*. 5.1%, p = 0.012), particularly due to APS events (18.5% *vs*. 1.9%, p <0.001) compared to the APS control, as well as the need for immune modulation or surgery.

Finally, we aimed to develop a tool that would assist clinicians in predicting TrAPS using the TrAPScore. The latter was based on a multivariate regression analysis using four parameters, each graded in regard to its relative impact: severe thrombocytopenia (4 points), heart valve disease (4 points), microvascular involvement (3 points), and triple-positive serology (2 points). Utilizing this score, values higher than 6 resulted in a positive predictive value of 78%–82.5%, while a value lower than 4 had a negative predictive value of 96.5%. Although this score requires further validation through additional studies, we believe it has the potential to provide a quantitative measure of a relatively common unmet clinical need. Whether this tool will effectively assist clinicians in navigating therapy for this challenging subset of patients remains to be determined.

The strength of both TrAPS and the TrAPScore is most evident when comparing this group to severe APS patients—the latter defined as those who experienced no more than one breakthrough event, required sporadic immunomodulation, or APS-related surgery but overall maintained disease control. In this context, differences in clinical presentation and antibody titers were less pronounced. Additionally, the commonly used severity index, aGAPSS, showed some differences; however, these distinctions may not be sufficient to definitively classify patients as having a truly refractory disease. As illustrated in the figure, the TrAPScore demonstrates a clear separation between patients with severe APS and those with TrAPS, highlighting its potential utility in distinguishing clinically untreatable cases.

This study is not free of limitations derived from its retrospective single-center design regarding patient selection and disease severity estimation. However, this approach enabled us to assess severity based on long-term follow-ups, and we applied three independent re-assessments of the clinical course for each patient to better define the risk factors. Second, the institution of a new severity subgroup is somewhat subjective in deciding what qualifies as severe, which requires further validation in different cohorts. Lastly, the fact that our center is a tertiary referral center might bias our APS cohort as patients with more severe disease are typically referred; this might be reflected by the relatively large group of cAPS in our cohort [4.6% *vs*. approximately 1% in previous reports (11)]. We believe that these biases are addressed with the design of this study, that is, objective criteria in patient selection, a homogeneous study group (primary thrombotic APS patients), active follow-up/monitoring of all patients, and multivariate statistical analysis. Nevertheless, this finding requires confirmation in future studies with different cohorts. Nonetheless, we hope that these findings will address a clinical need and be used by other clinicians to evaluate the accuracy of our findings.

## Conclusion

This study evaluated a new subset of resistant thrombotic APS disease, termed “terrible APS”—TrAPS. This new variant of APS was defined by the presence of multiple breakthrough thrombotic events despite proper anticoagulation and/or the need for immune-mediated treatments and/or requirement for APS-associated surgical intervention.

The TrAPScore was used to assess this variant of the disease based on four parameters: severe thrombocytopenia (4 points), heart valve disease (4 points), microvascular involvement (3 points), and triple positive aPLs (2 points). Based on our data, a TrAPScore higher than 6 had a positive predictive value of 78%–82.5%, whereas a value lower than 4 had a negative predictive value of 96.5%. Future studies are needed to determine whether this score truly differentiates cases that are resistant to most current treatments and will require reassessment of the management of these cases.

## Data Availability

The original contributions presented in the study are included in the article/supplementary material. Further inquiries can be directed to the corresponding author.
